# Impaired hippocampal representation of place in the *Fmr1*-knockout mouse model of fragile X syndrome

**DOI:** 10.1038/s41598-018-26853-z

**Published:** 2018-06-11

**Authors:** Tara Arbab, Cyriel M. A. Pennartz, Francesco P. Battaglia

**Affiliations:** 1 Cognitive and Systems Neuroscience, Swammerdam Institute, Center for Neuroscience, Faculty of Science, University of Amsterdam, Science Park 904, 1098 XH Amsterdam, The Netherlands; 2 Netherlands Institute for Neuroscience, Institute of the Royal Netherlands Academy of Arts and Sciences, Meibergdreef 47, 1105 BA Amsterdam, The Netherlands; 3 Department of Psychiatry, Academic Medical Center, University of Amsterdam, Postal Box 22660, 1100 DD Amsterdam, The Netherlands; 4 Research Priority Program Brain and Cognition, University of Amsterdam, Postal Box 94216, 1090 GE Amsterdam, The Netherlands; 5 Donders Institute for Brain, Cognition, and Behaviour, Radboud Universiteit Nijmegen, Heyendaalseweg 135, 6525 AJ Nijmegen, The Netherlands

## Abstract

Fragile X syndrome (FXS) is an X-chromosome linked intellectual disability and the most common known inherited single gene cause of autism spectrum disorder (ASD). Building upon demonstrated deficits in neuronal plasticity and spatial memory in FXS, we investigated how spatial information processing is affected *in vivo* in an FXS mouse model (*Fmr1*-KO). Healthy hippocampal neurons (so-called place cells) exhibit place-related activity during spatial exploration, and their firing fields tend to remain stable over time. In contrast, we find impaired stability and reduced specificity of *Fmr1*-KO spatial representations. This is a potential biomarker for the cognitive dysfunction observed in FXS, informative on the ability to integrate sensory information into an abstract representation and successfully retain this conceptual memory. Our results provide key insight into the biological mechanisms underlying cognitive disabilities in FXS and ASD, paving the way for a targeted approach to remedy these.

## Introduction

The most common known inherited single gene cause of autism spectrum disorder (ASD) is fragile X syndrome (FXS)^1,2^: an intellectual disability in which expression of the fragile X mental retardation protein (FMRP) is silenced or affected by loss of function mutations, resulting in disturbed neuronal communication^3,4^. Due to its simple genetic etiology, FXS shows promise for understanding neuropsychiatric disease from genes, to circuits, to cognitive impairment^5^. Particularly affected in FXS human patients and animal models is the hippocampus^6,7^, a brain structure essential for consolidating experiences into conceptual and spatial memory^8–11^. When healthy humans and animals explore a space, hippocampal neurons (so-called place cells) exhibit place-related activity^12,13^. These cells tend to stably maintain their firing fields over time, forming a spatial representation of the environment^14,15^. It follows that anomalous place cell activity in disease models may be characteristic of cognitive impairment in neurological disorders. We used a spatial exploration paradigm to investigate in an FXS mouse model (*Fmr1*-KO)^16,17^ how spatial information processing is affected *in vivo* by recording hippocampal place cell activity.

## Results and Discussion

### Unaffected exploratory behavior in Fmr1-KO mice

**Figure 1:**
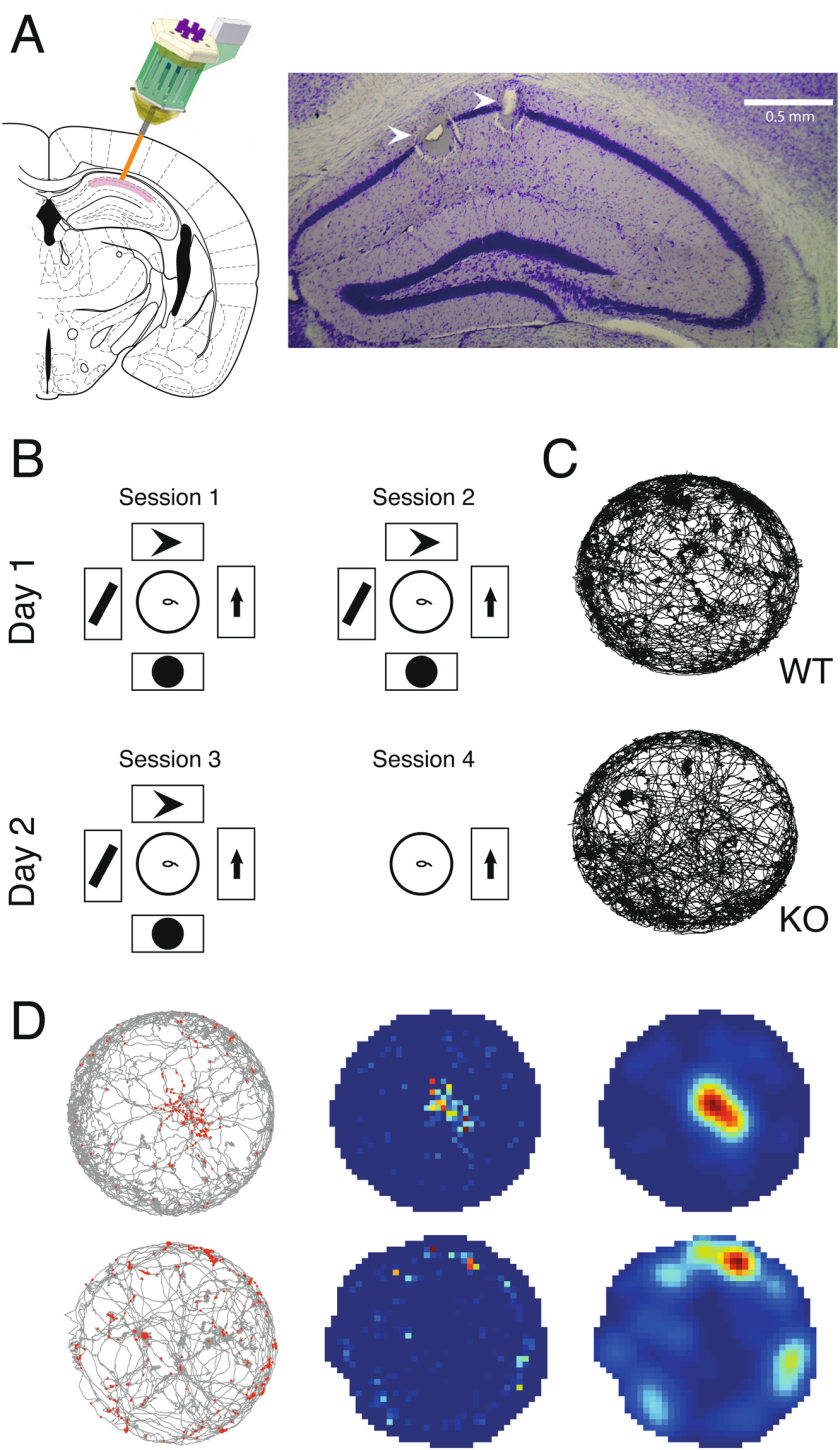
Experimental Setup. (**A**) Histology. Left, schematic of microdrive implantation target, modified from^50^. Right, coronal section showing the recording locations of two tetrodes in dorsal hippocampus CA1 (arrowheads). (**B**) Schematic of the behavioral protocol. On two consecutive days, in two sessions per day, animals freely explored a circular open field arena (middle). The arena was surrounded by four posters of geometric figures in the first three sessions, and only one poster in the fourth session. (**C**) Behavior. Accumulated trajectories of a WT and a KO animal exploring the arena during an example session. (**D**) Illustration of the three steps of firing map construction, for an example WT place cell (top row) and an example KO place cell (bottom row). Left, accumulated trajectory of animal exploration during the session, with spikes recorded from a single pyramidal cell superimposed in red. Middle, heat map of these spikes created by binning and normalizing this data. Right, smoothed heat map of these binned and normalized spikes.

We recorded neuronal activity in hippocampal CA1 (Fig. 1A) during four subsequent spatial exploration sessions (across two days) in five *Fmr1*-KO mice and five WT control mice (Fig. 1B). The first two recording sessions (on the morning and afternoon of the first day) and the third recording sessions (on the morning of the second day) were done with a complete set of four visual cues marking the environment (“Full cue” sessions). For the fourth session (on the afternoon of the second day), three of these visual cues were removed from the room, leaving an incomplete set of cues by which the animal could localize itself (“Probe” session). Our recordings yielded 124 WT and 141 *Fmr1*-KO putative pyramidal cells that exhibited spatially modulated activity (place cells). The numbers of place cells recorded per animal and their contribution to the total number of cells recorded per genotype are provided in Table S1.

Hippocampal place fields are thought to result from a complex process of integration of different types of information during exploration of an environment. Instantaneous multisensory inputs from the environment are combined into a ‘scaffold’ of internally generated representations, which depend to a large extent on self-motion information^18,19^. Therefore, we first controlled for parameters of exploratory behavior, which may act as a confounding factor for neuronal processing of spatial information (Fig. 1C). We found no difference between WT and *Fmr1*-KO running speed (WT median = 5.9 cm/s n = 30, KO median = 5.7 cm/s n = 33, Mann-Whitney *U* = 422, P = 0.32 two-tailed) and thigmotaxis (average distance from the center of the arena, WT median = 25.62 cm n = 38, KO median = 25.18 cm n = 32, Mann-Whitney *U* = 494, P = 0.18 two-tailed) across sessions. Additionally, there was no difference in the maximum and mean pyramidal cell firing rate across genotypes (Table S2).

### Reduced spatial specificity of place cells in Fmr1-KO mice

Pyramidal cells of both genotypes exhibited spatially selective activity: place fields (Fig. 1D). We found no difference between WT and *Fmr1*-KO mice in the number of place fields per cell, or the spatial information that each spike carried. However, *Fmr1*-KO place fields were significantly larger than those of wildtype animals, based both on counting active pixels in the normalized map (WT median = 9448 n = 123, KO median = 9603 n = 141, Mann-Whitney *U* = 5666, P < 0.0001 two-tailed) and by comparing the place fields in the smoothed maps (Table S2).

**Figure 2:**
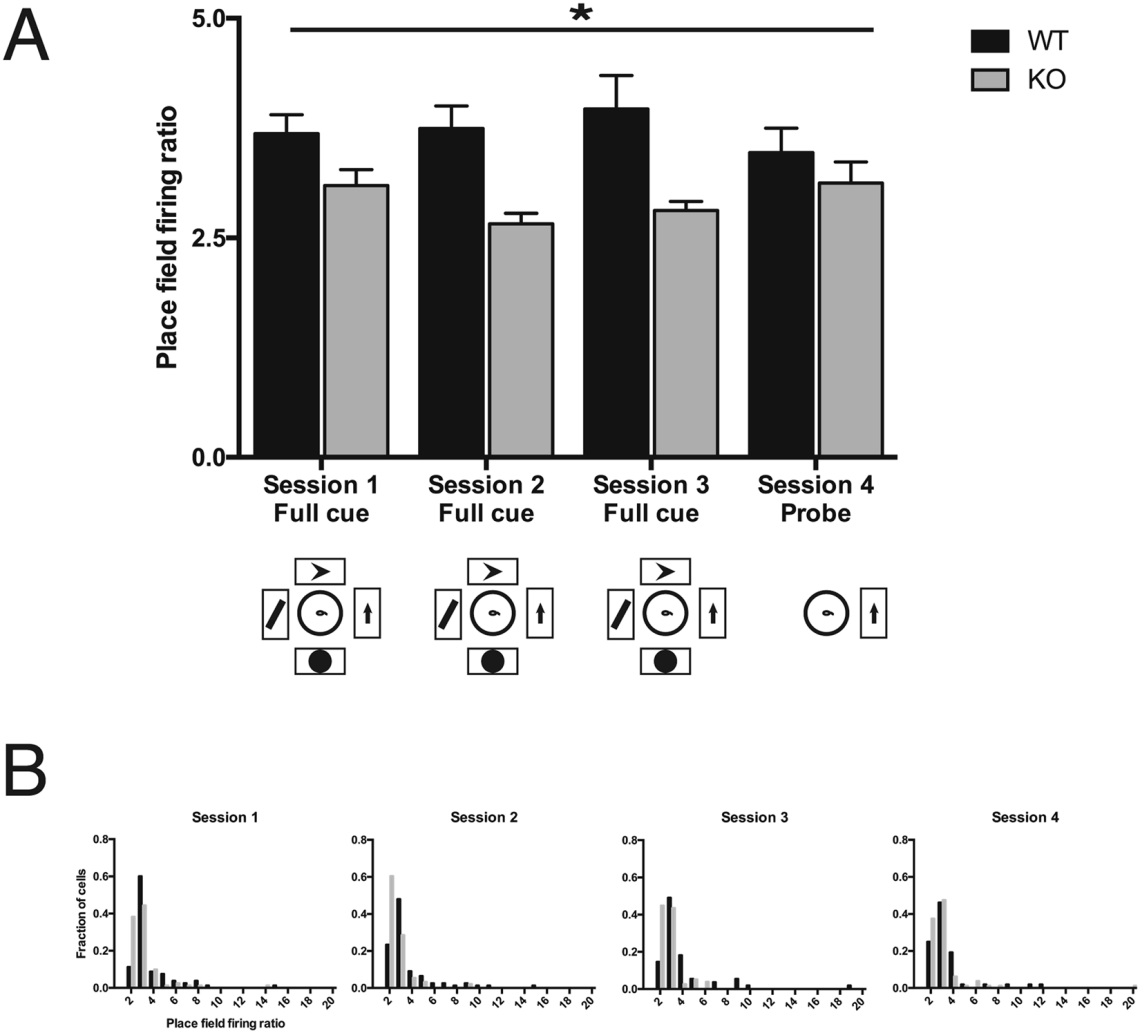
Spatial specificity of place cells per session. (**A**) Place field firing rate increase of WT (black) and *Fmr1*-KO (gray) pyramidal cells within their respective fields, relative to the firing rate of each cell outside its field (the place field firing ratio). Data are represented as mean ± SEM. Session 1: WT 80 cells, mean = 3.69, SEM = 0.22; KO 81 cells, mean = 3.09, SEM = 0.18. Session 2: WT 77 cells, mean = 3.75, SEM = 0.25; KO 91 cells, mean = 2.66, SEM = 0.12. Session 3: WT 55 cells, mean = 3.97, SEM = 0.38; KO 78 cells, mean = 2.81, SEM = 0.10. Session 4: WT 52 cells, mean = 3.47, SEM = 0.28; KO 80 cells, mean = 3.12, SEM = 0.24. (**B**) Distributions of place field firing rate increase of WT (black) and *Fmr1*-KO (gray) pyramidal cells within their respective fields, relative to the firing rate of each cell outside its field (the place field firing ratio). *P < 0.0001 main effect of genotype (two-way genotype x session ANOVA: F_1,586_ = 25.55). There was no effect of session and no interaction effect.

We determined spatial specificity of place cells as the firing rate increase of each cell within its field, relative to the firing rate of the same cell outside its field (Fig. 2). Spatial specificity of *Fmr1*-KO pyramidal cells was significantly reduced compared with WT (two-way genotype x session ANOVA, effect of genotype: F_1,586_ = 25.55; P < 0.0001). There was no effect of session and no interaction effect across cells.

### Impaired short-term stability of spatial representation in Fmr1-KO mice

**Figure 3:**
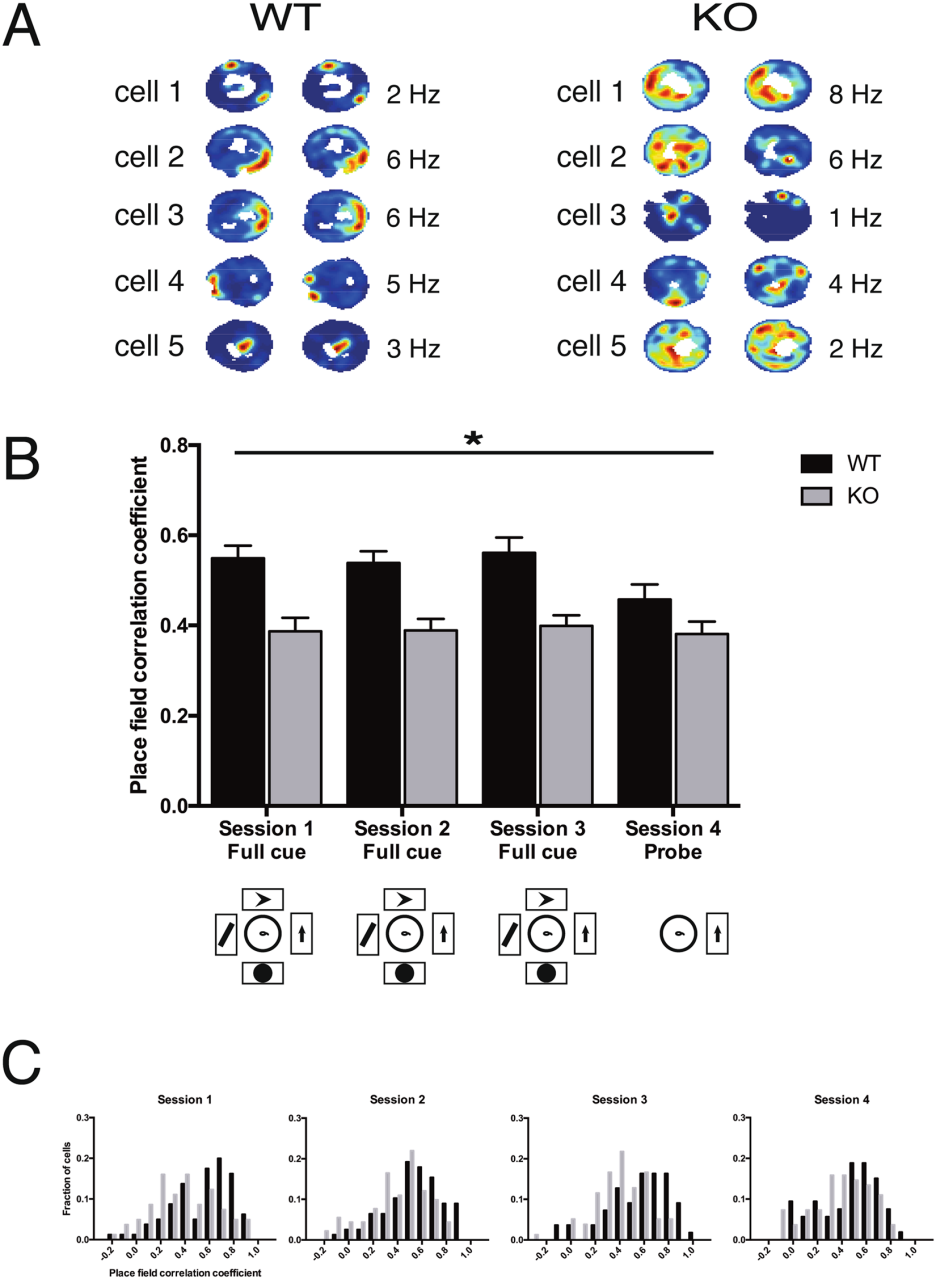
Stability of firing rate maps within sessions. (**A**) Example WT and KO firing rate maps (selected across all mice and sessions), split between the first (left panels) and second (right panels) halves of each recording session, to illustrate the stability of each map. Each heat map is scaled by the maximum firing rate (indicated in Hz) of the cell within that session. Areas of the arena that were not visited during the recording session are marked in white. (**B**) Correlation of WT (black) and *Fmr1*-KO (gray) firing rate maps. Data are represented as mean ± SEM. Session 1: WT 80 fields, mean = 0.55, SEM = 0.028; KO 81 fields, mean = 0.39, SEM = 0.03. Session 2: WT 78 fields, mean = 0.54, SEM = 0.03; KO 91 fields, mean = 0.39, SEM = 0.03. Session 3: WT 55 fields, mean = 0.56, SEM = 0.03; KO 78 fields, mean = 0.40, SEM = 0.02. Session 4: WT 53 fields, mean = 0.46, SEM = 0.03; KO 82 fields, mean = 0.38, SEM = 0.03. (**C**) Distributions of the stability of WT (black) and *Fmr1*-KO (gray) pyramidal cells. *P < 0.0001 main effect of genotype (two-way genotype x session ANOVA: F_1,590_ = 46.34). There was no effect of session and no interaction effect.

To assess the stability of place cell activity, we first evaluated the similarity of the firing rate maps between the halves of each session (Fig. 3). Pixel-based Pearson correlations between rate maps were significantly reduced in *Fmr1*-KO mice compared with WT (two-way genotype x session ANOVA, effect of genotype: F_1,590_ = 46.34; P < 0.0001). We found no effect of session and no interaction effect. Comparison between the quarters of each session yielded the same conclusion: correlations between rate maps were significantly reduced in *Fmr1*-KO mice compared with WT (two-way genotype x session ANOVA, effect of genotype: F_1,1457_ = 92.58; P < 0.0001) with no effect of session and no interaction effect (Table S3).

Since each animal was used in multiple consecutive experiments in which only the wall cues were changed and the arena stayed the same, we assessed whether the novelty of the environment was diminished between experiments. We compared place field stability on the first (novel) day of each consecutive experiment and found no increase, allowing us to rule out the possibility that the mice were using subtle intra-maze cues to skew the stability of spatial representation between experiments.

Thus, the effects of the FXS mutation on spatial representation in *Fmr1*-KO mice are not attributable to behavioral differences or basic physiological properties such as pyramidal cell firing rate. However, the relative strength of firing of WT place cells within fields is greater than in *Fmr1*-KO mice, the latter shows increased size of place fields, and the location of place responses is less stable in *Fmr1*-KO mice than in WT controls in short intervals within recording sessions in the same environment.

As for any neural integration operation, self-localization is affected by the accumulation of errors, resulting in drift which increases with time^20,21^. Spatially informative sensory cues can realign the drifting map, therefore reducing error. In interpreting our current findings, one possibility is that the FXS mutation affects the sensory information-dependent updating of the self-motion based map, while leaving the map itself relatively spared.

Another possibility is that the *Fmr1*-KO mice are paying less attention to the cues, so that their information is not integrated properly into the spatial representation. As sensory cues may rapidly induce profound changes in the spatial map^22^, in an attention modulated way^15^, the increased instability we observe in *Fmr1*-KO mice may be due to a lower weight of sensory inputs in determining place cell firing. Indeed, FXS patients show defective attention and integration of new information^23^.

### Fmr1-KO spatial representation does not reflect changes in environment

**Figure 4:**
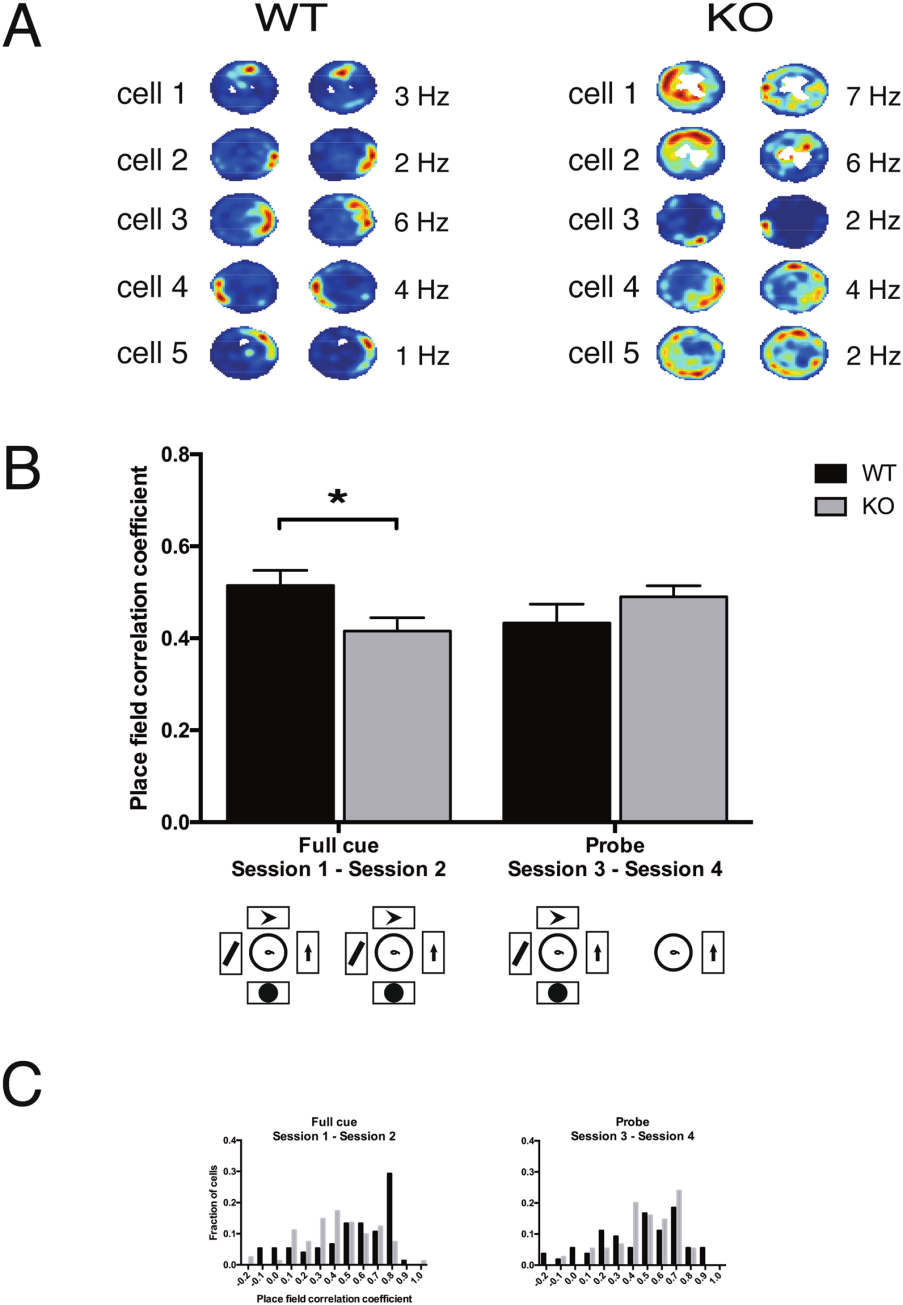
Stability of firing rate maps between sessions. (**A**) Example WT and KO firing rate maps (selected across all mice and sessions), split between the first (left panels) and second (right panels) daily recording sessions, to illustrate the stability of each map. Each heat map is scaled by the maximum firing rate (indicated in Hz) of the cell within that session. Areas of the arena that were not visited during the recording session are marked in white. (**B**) Correlation of WT (black) and *Fmr1*-KO (gray) firing rate maps. Data are represented as mean ± SEM. Full cue session: WT 75 fields, mean = 0.51, SEM = 0.03; KO 81 fields, mean = 0.42, SEM = 0.03. Probe session: WT 54 fields, mean = 0.43, SEM = 0.04; KO 75 fields, mean = 0.49, SEM = 0.02. (**C**) Distributions of the stability of WT (black) and *Fmr1*-KO (gray) pyramidal cells. *P < 0.05 (Bonferroni’s multiple comparisons test).

To determine whether *Fmr1*-KO mice are impaired in sensory information-dependent updating of their spatial representation, we assessed the stability of firing rate maps between “Full cue” and “Probe” sessions on the same day. Here, we found no direct effect of genotype or session, but we found a significant interaction effect (two-way genotype x session ANOVA followed by Bonferroni’s multiple comparisons test, interaction effect: F_1,281_ = 6.074; P = 0.0143). Specifically, *Fmr1*-KO pyramidal cells showed a significantly reduced spatial correlation of activity compared with WT between the “Full cue” sessions (i.e., sessions 1 and 2, in which the open field arena was surrounded by the same four visual cues). The firing rate map correlation between the “Full cue” (i.e., sessions 1 and 2) and the novel “Probe” (i.e., sessions 3 and 4, in between which three of these cues were removed) environment however, did not significantly differ between genotypes (Fig. 4), possibly signifying that constancy of external cues is not the main factor driving place cells in *Fmr1*-KO, which might rely on other (e.g. self-motion or internal^24^) sources of information.

### Spatial representation impairments in Fmr1-KO provide biomarker for FXS deficits

Both animal^8,9^ and human^10,11^ studies link the hippocampus to spatial, contextual, and autobiographical memory. In the same way that place cells in animals exploring an environment can encode that space, the activity of hippocampal neurons in humans can encode abstract representations of multi-sensory perceptual information^13,25^. Hippocampal dysfunction is a critical component of intellectual pathologies such as FXS and ASD, in which impairments of conceptualization and memory are observed^6,7,26^. Here, the delicate system that allows the brain to carefully fine-tune which information it retains is disrupted, because of the devastating effect on activity-dependent synaptic plasticity that underlies learning and memory. This ultimately contributes to anomalous processing of social and environmental cues and associated deficits in memory and cognition^27^. Although they are equally affected neurologically, it has been difficult to assess these cognitive deficits in animal models with the same robustness as in human FXS patients^28^. Our findings take the middle ground by demonstrating on a cellular level *in vivo* that altered physiology in *Fmr1*-KO leads to impaired hippocampal information processing.

Indeed, one should note that place field disruption is not unequivocally correlated with a spatial and/or navigation memory deficit. In fact, it has been shown that even subtler distortions of hippocampal activity, for example ones affecting temporal coding, but leaving the place field map intact, may also correlate with learning and memory deficits^29–31^. On the other hand, reports from rat virtual reality experiments suggest that while place fields are disrupted, animals are still capable of learning spatial tasks in virtual space^32^. Similarly, mice with a deleted GluA1 subunit of the AMPA receptor have impaired place fields, but can solve a spatial memory task (while being impaired in a working memory task)^33^. Still, our finding of impaired stability of place fields in *Fmr1*-KO mice suggests deeply disrupted hippocampal function, which predicts cognitive deficits associated with FXS, especially as one moves to the more delicate, demanding cognitive abilities affected in human FXS patients.

There is a wide array of FXS physiological deficits which might underlie our results. Stability of spatial representation requires long-term potentiation (LTP) associated with NMDA receptor activity in hippocampal CA1^34–37^. FMRP regulates subunit composition of hippocampal NMDA receptors^38^ and may therefore contribute to *Fmr1*-KO pathophysiology by affecting synaptic plasticity through altered subunit composition of NMDA receptors. Indeed, LTP deficits are observed in *Fmr1*-KO mice^39–42^. Additionally, *Fmr1*-KO mice show higher dendritic expression of the *HCN1* gene in hippocampal CA1^43^, which might limit certain aspects of spatial memory and plasticity in pyramidal neurons by affecting the ability of the entorhinal cortex to excite them^44^. The instability we find in *Fmr1*-KO firing rate maps may be interpreted within this context as an increase in *HCN1*-mediated control over CA1 pyramidal cell plasticity from entorhinal inputs through the perforant pathway, which affects the sensory information-dependent updating of the self-motion based map as described above. Finally, disrupted network mechanisms^45^ regulating the inflow of information between the hippocampus and entorhinal cortex^46,47^ may contribute to improper routing of sensory information to the hippocampus, or in the failure to elicit spike-timing dependent plasticity^37,48,49^. While the cognitive effects of these deficits have proven difficult to assess behaviorally in *Fmr1*-KO^28^, we find that they may contribute to disrupting neural mechanisms that establish associations between external cues and internally generated or self-motion dependent representations.

Hippocampal place cells are one of the best understood systems in the brain where we have reached an initial understanding of the relationship between neural dynamics, information encoding, and cognition. Here we have shown that they may provide a powerful tool in understanding intellectual disability and ASD in a mouse model of FXS, in which it has been surprisingly difficult to demonstrate consistent cognitive deficits despite its clear genetic etiology. We find impaired specificity and stability of CA1 place cell activity in *Fmr1*-KO mice, both within and across subsequent exploration sessions, while these mice show a relatively spared place field response and their behavior and firing-rate parameters do not significantly differ from WT mice. Our results link impaired physiology with cognition more deeply than possible with traditional behavioral of physiological assays, and offer a potential biomarker for testing of therapeutic strategies.

## Methods

### Subjects

We used five *Fmr1*-KO mice^17^ and five littermate wildtype (WT) control mice. All experiments were performed in accordance with Dutch National Animal Experiments regulations, were approved by the Universiteit van Amsterdam, and were carried out by certified personnel. Animals were received from the Erasmus Medisch Centrum Rotterdam breeding unit at an age of 8 weeks and group-housed until surgery. They were maintained on a regular 12-hour light-dark cycle (lights on: 8 am, lights off: 8 pm) and received standard food pellets and water *ad libitum* throughout the experiment. To minimize bias due to possible undetected changes in environmental conditions, *Fmr1*-KO and WT animals were always studied in pairs; both recordings were done on the same day and counterbalanced per genotype. Once habituated to the experimenter and handling, the mice underwent drive implantation surgery under buprenorphine-isoflurane anesthesia and were left to recover fully before the start of the experiment.

### Electrophysiological techniques

Six independently moveable tetrodes were loaded into a custom-made microdrive^37,50^ and implanted over the dorsal hippocampus (AP: −2.0 mm, ML: −2 mm^51^; Fig. 1A). The tetrodes were lowered into the CA1 pyramidal cell layer guided by electrophysiological signals (sharp wave-ripple events) over the course of days following implantation surgery. Electrophysiological activity was recorded on a 27-channel analog Neuralynx data acquisition system at a 32 kHz sampling rate. Tetrode signals (bandpass filtered 0.6–6.0 kHz) were referred to a nearby tetrode which was targeted to a location devoid of single unit activity. Single-unit data were preprocessed with Klustakwik^52^ for automated spike clustering and the results were manually refined using Klusters^53^. The resulting spike trains were analyzed using custom-written MATLAB code. Animal tracking position was extracted from video footage by Ethovision XT software (Noldus, Wageningen, the Netherlands) which was synchronized with the electrophysiology data acquisition system. At the end of experiments, electrolytic lesions were made to verify tetrode placement. Brain tissue was fixed by transcardial perfusion and Nissl stained (Fig. 1A). Only animals with clear lesions in the CA1 pyramidal layer were included in the analysis.

### Behavioral protocol

An experiment consisted of four sessions (two per day on two consecutive days) during which hippocampal neural ensemble activity was recorded as the mice freely explored (without foraging for food) a fully transparent, circular open field arena (diameter 64 cm) for 30 min. The arena was surrounded by black curtains and four large posters of geometric figures as visual cues (Fig. 1B). In the final (fourth) session, three of the visual cues were removed (“Probe” session); the same cues were removed for both genotypes. The two daily recording sessions were separated by a two-hour break, during which the animal rested in its home cage. Each animal was screened in its home cage in the experiment room for 30 min prior to each recording. Each animal was used for multiple (consecutive) experiments (on average 3 experiments per animal). A new set of visual wall cues was selected for each iteration: session 1 was always the first recording in the novel environment.

### Neuronal analysis

Periods of inactivity (animal speed <3 cm/s) were excluded from analysis. Videotracking data were visually inspected, checked for accuracy, and corrected manually when necessary. Recording stability of individual clusters of spikes was examined; clusters whose first principal component drift exceeded more than three standard deviations across both sessions within a day were excluded from analysis. Classification of putative pyramidal cells was based on their firing rate and the mean of the autocorrelogram, as previously described by our lab^37^.

### Place cell analysis

To create firing maps of individual neurons, spike data were (1) plotted on binned arena occupancy data (pixels: 2 × 2 cm), (2) normalized by the total time spent in each bin, and (3) smoothed (radius: 2). These three steps are illustrated for two example WT place cells recorded in two separate sessions in Fig. 1D. Bins that received insufficient sampling (<200 ms) were excluded from analysis. Only neurons that displayed place-related activity in at least one session were included in analysis. Place fields were defined as areas larger than 10 adjacent pixels where a pyramidal cell exhibited more than 30% of its maximum firing rate. Spatial information per spike was calculated as described in^54^. Spatial specificity (the place field firing ratio) was calculated as the firing rate increase of each cell within its field, relative to the firing rate of the same cell outside its field (in-field firing rate divided by out-field firing rate).

### Data availability

The dataset generated and analyzed in the current study is available from the corresponding author on reasonable request.

## Electronic supplementary material

Supplementary tables
