# Supplementary material for: Impaired hippocampal representation of place in the Fmr1-knockout mouse model of fragile X syndrome
Source: Sci Rep. 2018 Jun 11;8:8889. doi: 10.1038/s41598-018-26853-z (PMC5995880; doi:10.1038/s41598-018-26853-z)
Supplement: Supplementary file 1 — Supplementary tables [file 41598_2018_26853_MOESM1_ESM.docx]

**Title**

Impaired hippocampal representation of place
in the *Fmr1*-knockout mouse model
of Fragile X syndrome

**Authors / Affiliations**

Tara Arbab^1,2,3^, Cyriel MA Pennartz^1,4^, Francesco P Battaglia^1,5^

^1^ Cognitive and Systems Neuroscience, Swammerdam Institute, Center for Neuroscience, Faculty of Science, University of Amsterdam, Science Park 904, 1098 XH Amsterdam, The Netherlands

^2^ Netherlands Institute for Neuroscience, Institute of the Royal Netherlands Academy of Arts and Sciences, Meibergdreef 47, 1105 BA Amsterdam, The Netherlands

^3^ Department of Psychiatry, Academic Medical Center, University of Amsterdam, Postal Box 22660, 1100 DD Amsterdam, The Netherlands

^4^ Research Priority Program Brain and Cognition, University of Amsterdam, Postal Box 94216, 1090 GE Amsterdam, the Netherlands

^5^ Donders Institute for Brain, Cognition, and Behaviour, Radboud Universiteit Nijmegen, Heyendaalseweg 135, 6525 AJ Nijmegen, the Netherlands

**Corresponding Author**

Tara Arbab, [tara.arbab@gmail.com](mailto:tara.arbab@gmail.com)

| **Genotype** | **Total number of cells** | **Animal** | **Number of cells** | **Proportion of total** |
| --- | --- | --- | --- | --- |
| **WT** | **124** | **1** | 17 | ~14% |
|  |  | **2** | 14 | ~11% |
|  |  | **3** | 13 | ~10% |
|  |  | **4** | 29 | ~23% |
|  |  | **5** | 51 | ~41% |
| **KO** | **141** | **1** | 12 | ~9% |
|  |  | **2** | 7 | ~5% |
|  |  | **3** | 46 | ~33% |
|  |  | **4** | 70 | ~50% |
|  |  | **5** | 6 | ~4% |

Table S1. Numbers of WT and *Fmr1*-KO hippocampal CA1 place cells.

Total number of putative pyramidal cells that exhibited spatially modulated activity (place cells) for each genotype and animal. Each animal’s contribution to the total number of cells recorded per genotype is presented as the percentage of recorded cells per genotype.

| **Descriptive statistics of WT and *Fmr1*-KO hippocampal CA1 place cells** | | **WT** Median | ***Fmr1*-KO** Median | **Mann-Whitney *U*** | **P value** |
| --- | --- | --- | --- | --- | --- |
| **Spike**  **parameters** | **Maximum firing rate (Hz)** | 3.8 | 4.0 | 8265 | n.s. |
|  | **Mean firing rate (Hz)** | 0.42 | 0.56 | 7511 | n.s. |
| **Spatial coding parameters** | **Number of place fields per cell** | 1 | 1 | 8350 | n.s. |
|  | **Place field size (as fraction of the arena)** | 0.21 | 0.37 | 6220 | 0.0001 |
|  | **Spatial information per spike** | 1.64 | 1.62 | 8553 | n.s. |

Table S2. Descriptive statistics of WT and *Fmr1*-KO hippocampal CA1 place cells.

Medians of WT and *Fmr1*-KO parameters of place cell spikes and spatial coding. Data were compared using a Mann-Whitney test.

| **Stability of firing rate maps within sessions** | **Between quarters** | **WT** Mean ± SEM | ***Fmr1*-KO** Mean ± SEM |
| --- | --- | --- | --- |
| **Session 1** | **1 and 2** | 0.43 ± 0.04 | 0.23 ± 0.04 |
|  | **2 and 3** | 0.48 ± 0.03 | 0.30 ± 0.04 |
|  | **3 and 4** | 0.47 ± 0.03 | 0.25 ± 0.04 |
| **Session 2** | **1 and 2** | 0.47 ± 0.04 | 0.27 ± 0.03 |
|  | **2 and 3** | 0.44 ± 0.04 | 0.29 ± 0.04 |
|  | **3 and 4** | 0.46 ± 0.04 | 0.30 ± 0.03 |
| **Session 3** | **1 and 2** | 0.48 ± 0.04 | 0.33 ± 0.03 |
|  | **2 and 3** | 0.47 ± 0.04 | 0.37 ± 0.03 |
|  | **3 and 4** | 0.48 ± 0.04 | 0.30 ± 0.03 |
| **Session 4** | **1 and 2** | 0.34 ± 0.04 | 0.29 ± 0.03 |
|  | **2 and 3** | 0.39 ± 0.04 | 0.30 ± 0.03 |
|  | **3 and 4** | 0.35 ± 0.05 | 0.32 ± 0.04 |

Table S3. Stability of firing rate maps within sessions.

Means and SEM of WT and *Fmr1*-KO firing rate map correlations between subsequent quarters of each recording session.
